# Patient experiences of tuberculosis treatment deferral after a trace Xpert Ultra result: a prospective cohort study

**DOI:** 10.1186/s40249-025-01338-0

**Published:** 2025-07-15

**Authors:** Caitlin Visek, James Mukiibi, Mariam Nantale, Annet Nalutaaya, Patrick Biché, Joowhan Sung, Francis Kayondo, Joab Akampurira, Michael Mukiibi, Rogers Kiyonga, Achilles Katamba, Emily A. Kendall

**Affiliations:** 1 Division of Infectious Diseases, Johns Hopkins University School of Medicine, 1830 E. Monument st, 4th Floor, Baltimore, MD 21205 USA; 2 Uganda Tuberculosis Implementation Research Consortium, Walimu, Unit 4, Plot 5-7, Coral Crescent, Kololo, P. O. Box 9924, Kampala, Uganda; 3 Johns Hopkins Bloomberg School of Public Health, 615 N. Wolfe St, Baltimore, MD 21205 USA; 4 Makerere University College of Health Sciences, Upper Mulago Hill, P.O. Box 7072, Kampala, Uganda

**Keywords:** Tuberculosis, Molecular diagnostic testing, Diagnostic uncertainty, Patient preference, Patient experience

## Abstract

**Background:**

A “trace” result from the Xpert Ultra molecular tuberculosis test indicates *Mycobacterium tuberculosis* DNA detection but may not always signify tuberculosis disease. Little is known about the experiences of individuals with trace results who are not immediately treated. We surveyed patients with trace results to better understand their experiences and preferences related to their uncertain tuberculosis status.

**Methods:**

We enrolled adults and adolescents with trace Xpert Ultra sputum results, plus individuals with positive (at a semiquantitative level greater than trace) results (“positive controls”) and with negative results (“negative controls”), from community-screening and clinic settings in Kampala, Uganda between February 2021 and December 2024. After an extensive clinical, laboratory, and radiographic evaluation, participants not recommended to start tuberculosis treatment immediately were closely monitored with interval reassessments. Starting in September 2021, surveys captured participants’ perceptions and preferences related to their uncertain tuberculosis status at baseline and one and six months later. We compared categorial variables using Pearson’s chi-squared test or Fisher’s exact test with a significance level of 0.05.

**Results:**

A total of 329 people with trace sputum (PWTS), 241 positive controls, and 279 negative controls were enrolled. Among PWTS surveyed, 22% (28/129) and 23% (30/129) thought they were likely to have or develop tuberculosis, respectively, and most reported low associated anxiety initially (80%, 263/329) and during follow-up. While 53% (174/329) would have favored treatment at baseline if not in the study, only 30% (41/136) of those who remained untreated were inclined toward treatment at six months. Participants chose a sensitive hypothetical test, even with high false-positivity risk, over one with lower sensitivity.

**Conclusions:**

Most PWTS in our study reported a low self-perceived likelihood of having or developing tuberculosis and low anxiety during follow up. Deferring treatment for PWTS is acceptable to most patients when sufficient testing and monitoring are available; in other contexts, upfront treatment may be preferable.

**Graphical Abstract:**

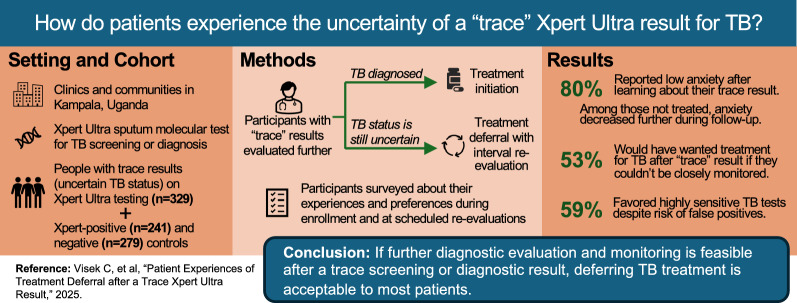

**Supplementary Information:**

The online version contains supplementary material available at 10.1186/s40249-025-01338-0.

## Background

Xpert MTB/RIF Ultra (“Xpert Ultra”; Cepheid) is a rapid molecular test widely used for diagnosing tuberculosis. Compared to the prior generation assay (Xpert MTB/RIF), Xpert Ultra is able to detect *Mycobacterium tuberculosis* (MTB) in sputum at lower bacillary burdens, through an improved cartridge design that allows for the processing of a larger volume sample and the addition of new multi-copy-per-genome gene targets [1]. However, results in the lowest category of positivity, termed “trace,” are often associated with negative sputum cultures and have been interpreted as false positive results [2–5]. Lacking reliable procedures for distinguishing between true- and false-positive trace results, current practice and World Health Organization guidance favor providing tuberculosis treatment to most people with trace sputum (PWTS) [6].

Still, for some PWTS who have mild or resolving illness (or who are asymptomatic) without other known evidence of tuberculosis disease, forgoing immediate treatment in favor of monitoring and repeat testing may be an acceptable, or even preferable, alternative. Tuberculosis treatment courses are often lengthy, lasting six months or more, and require multiple medications with significant potential for adverse reactions; studies have found that as many as 79% of patients receiving tuberculosis treatment experienced at least one adverse drug reaction [7, 8]. Patients with tuberculosis are also known to face steep social and economic burdens [9], which makes diagnosing patients with tuberculosis a consequential decision.

While avoiding overdiagnosis may be desirable, little is known about how patients might experience decisions to forgo immediate treatment or discussions about diagnostic uncertainty. In other clinical contexts, people with tuberculosis can experience emotional distress related to fear of stigma, disease transmission, and death [10–12]. If patients are recommended to undergo monitoring rather than upfront treatment, they must contend with uncertainty regarding their tuberculosis status as well as the time and inconvenience of additional diagnostic evaluation. No studies to date have evaluated whether such an approach adversely impacts patients’ wellbeing. To better understand the subjective experience of treatment deferral, we surveyed cohorts of PWTS who were not immediately treated for tuberculosis about their experiences, perceptions, and preferences related to their trace result and subsequent management. We also compared their responses to those of people with more certain tuberculosis statuses.

## Methods

### Study design and population

PWTS, and individuals with positive and negative Xpert Ultra results who served as controls, were identified through both community-based screening and clinic-based diagnostic evaluations in Kampala, Uganda between February 2021 and December 2024. Community-based screening occurred in high-risk areas without preceding screening tests. Clinic-based testing was typically symptom-driven, and Xpert Ultra was the standard test for pulmonary tuberculosis in the participating clinics. In both settings, expectorated sputum samples were tested for tuberculosis with Xpert Ultra, and all PWTS were contacted for enrollment. For most of the study period we also recruited controls: “negative controls” with negative Xpert Ultra results (matched approximately 1∶1 with enrolled PWTS in each setting), and “positive controls” with positive results at a level greater than trace (enrolled universally from community screening where approximately half of all positive results were trace, and matched 1∶1 in the clinic setting). Eligible participants were at least 15 years old and were not receiving tuberculosis treatment at the time of Xpert Ultra testing; clinic-recruited participants also had not received treatment for tuberculosis within the preceding 90 days. For PWTS, the informed consent process explained that the trace result “does not provide a clear answer about whether or not you have tuberculosis,” that it might indicate tuberculosis with a low bacterial burden or might be falsely positive, and that the study would include additional testing and follow-up to determine tuberculosis status and treatment needs.

All consenting participants underwent a comprehensive clinical, laboratory, and radiographic evaluation at baseline, including standardized interview, chest x-ray (CXR) and computed tomography (CT), medical history and physical examination, and laboratory testing including repeat sputum Xpert Ultra, sputum culture, tuberculosis immunoreactivity [QuantiFERON] testing, tuberculosis tongue swab molecular testing [13], HIV testing, and serum C-reactive protein (CRP) measurement, as well as urine lipoarabinomannan (LAM) and CD4 count measurement for participants living with HIV.

At baseline and on an ongoing basis, an independent physician review panel evaluated available clinical, laboratory, and radiographic data from PWTS and recommended treatment initiation whenever they judged that a given patient was highly likely to have tuberculosis or at high risk for harm from continued treatment deferral. Study-unaffiliated clinicians who cared for study participants could also initiate treatment at any time. For the current analysis, we consider a tuberculosis diagnosis to be microbiologically confirmed if the participant had either a sputum culture positive for MTB or a repeat Xpert Ultra result that was positive at a level greater than trace. Clinicians sometimes made additional TB diagnoses based on a combination of symptoms, risk factors, and/or imaging findings. Participants not recommended for treatment after their baseline evaluations underwent longitudinal monitoring with periodic repeat clinical, laboratory, and imaging evaluations (initial follow-up visits scheduled at one, three, and six months). Negative-control participants were reevaluated less frequently (first follow-up visit at six months). Positive-control participants were referred for tuberculosis treatment and had no further study follow-up.

The study was approved by the Makerere University School of Public Health Research and Ethics Committee (Protocol 901) and the Johns Hopkins Medicine Institutional Review Board (IRB00269370).

### Patient experience survey

Beginning on September 30, 2021, a structured questionnaire about subjective experiences and preferences was administered to participants at study enrollment and at one- and six-month follow-up visits (Appendix A). Questions asking participants to rate aspects of their experience used three- to six-level Likert scales. The questionnaire was developed in English, piloted and refined among Ugandan research staff, and professionally translated to Luganda. Research staff accessed the survey and captured responses using an electronic tool with conditional branching and completeness checks and received standardized training on survey administration.

The baseline questionnaire was administered after participants had been informed of their initial screening or diagnostic Xpert Ultra results and enrolled into the study. PWTS were asked how anxious they felt about the possibility of having or developing tuberculosis and whether they would be inclined to seek out tuberculosis treatment if they were not being offered additional testing and monitoring via the study. Those enrolled from the community were also asked to rate whether, considering everything they knew at the time, they believed themselves to currently have tuberculosis, and whether they thought themselves likely to develop tuberculosis in the future.

The baseline survey also included a series of vignette-based questions aimed at evaluating tradeoffs between sensitivity and specificity. All participants were asked to choose between alternative tuberculosis tests to recommend to their friends and family. One hypothetical test (representing high sensitivity but poor specificity) was labeled as “too strong” and described as correctly identifying everyone with tuberculosis but producing one false-positive result for every tuberculosis case correctly identified. The other (representing low sensitivity but excellent specificity) was labeled as “too weak” and described as generating no false-positive results but misclassifying one out of every two people who truly have tuberculosis as negative. Participants who chose the initial version of the “too strong” test were also presented with a follow-up scenario in which the number of false positives per true positive increased five-fold.

At the one-month (for PWTS) and six-month (for PWTS and negative controls) follow-up visits, participants who remained off tuberculosis treatment were surveyed again. They were asked to rate how inconvenient or unpleasant they had found completing each of the diagnostic tests they completed previously, and how valuable they considered each of the tests. Survey questions about anxiety, treatment inclination, and self-perception of tuberculosis status were also repeated.

### Statistical analysis

All analyses were performed with R statistical software version 4.3.1 (R Foundation for Statistical Computing, Vienna, Austria) and RStudio version 2023.06.1 + 524 (Posit Software, PBC, Boston, USA). Continuous variables were reported as median (interquartile range), and binary variables were reported as *n*/*N* (%). Categorial variables were compared using Pearson’s chi-squared test or Fisher’s exact test with a significance level of 0.05, and 95% confidence intervals were calculated using the Wilson score interval with continuity correction.

## Results

### Study enrollment and treatment initiation

**Fig. 1:**
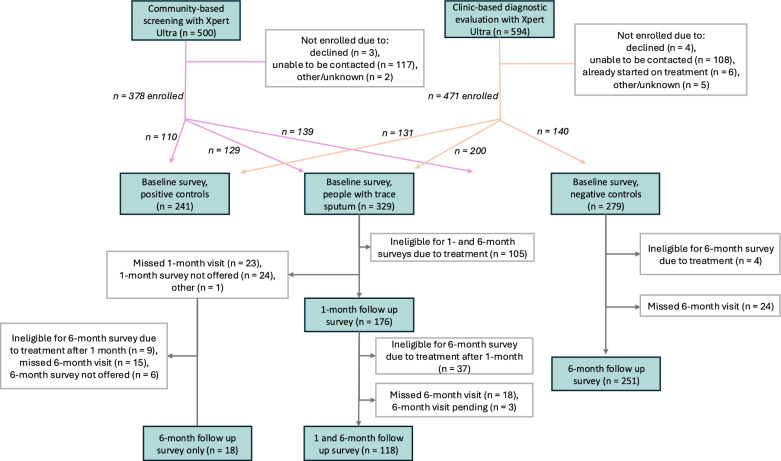
Overview of Xpert Ultra testing, participant enrollment, survey completion, and reasons for exclusion. Study participants enrolled based on Xpert Ultra result as positive controls (Xpert Ultra positive > trace), people with trace sputum (Xpert Ultra trace), or negative controls (Xpert Ultra negative). Surveys administered at baseline for all participants, at one month follow up visit for people with trace sputum, and at six month follow up visit for people with trace sputum and negative controls

A total of 849 participants were enrolled during the period included in this analysis: 378 recruited from community-based screening events and 471 from clinic settings (Fig. 1). These included 329 PWTS, 241 positive controls, and 279 negative controls (Table 1). Of the 329 enrolled PWTS, 105 (32%) started treatment prior to the one-month follow up visit, and 151 (46%) by the six-month visit.

**Table 1:** Recruitment setting and baseline demographic, clinical, and laboratory characteristics for study participants, stratified by study arm

Variable	People with trace sputum (*n* = 329)	Negative control participants (*n* = 279)	Positive control participants (*n* = 241)
Recruited through community screening [*n* (%)]	129 (39%)	139 (50%)	110 (46%)
Recruited after routine diagnostic testing [*n* (%)]	200 (61%)	140 (50%)	131 (54%)
Age in years [median (Q1, Q3)]	34 (27, 42)	34 (26, 40)	34 (28, 42)
Sex: Male [*n* (%)]	193 (59%)	161 (58%)	161 (67%)
Living with HIV [*n* (%)]	108 (33%)	74 (27%)	82 (34%)
CD4 < 200 cells/mm^3^ [*n* (%)]^1^	25/93 (27%)	*Not measured*	*Not measured*
Taking antiretroviral therapy [*n* (%)]^2^	74/104 (71%)	66/71 (93%)	43/80 (54%)
Tuberculosis symptoms present at enrollment [*n* (%)]^3^	287 (87%)	169 (61%)	233 (97%)
History of prior tuberculosis [*n* (%)]	99 (30%)	40 (14%)	40 (17%)
C-reactive protein, mg/L [median (Q1, Q3)]	3 (< 2.5, 14)	< 2.5 (< 2.5, 4)	41 (4, 104)

^*^Q1 = 25th percentile; Q3 = 75th percentile

^1^CD4 count missing for 15 participants with trace sputum living with HIV and not measured for negative or positive controls

^2^Among participants living with HIV, antiretroviral therapy status was missing for 4 people with trace sputum, 3 negative control participants, and 2 positive control participants

^3^Defined as having at least one of the following: cough lasting 2 or more weeks, hemoptysis, fever or chills, night sweats, weight loss

### Perceived tuberculosis risk, anxiety, and treatment inclination

At baseline, a majority of PWTS (80%, 263/329) reported little to no anxiety at baseline about the possibility that they could have or develop tuberculosis, while 15% (50/329) reported moderate anxiety and 5% (16/329) reported feeling very anxious. Anxiety was lowest in the community setting (*P* < 0.01). About half of participants in both settings reported that they would be inclined to start tuberculosis treatment were they not enrolled in the study (Table 2). For PWTS enrolled from the community setting, who were asked about perceived likelihoods of having or developing tuberculosis, a minority perceived themselves as likely to have tuberculosis currently (22%, 28/129) or develop it in the future (23%, 30/129). Fewer negative controls than PWTS from the community perceived themselves as likely to have tuberculosis currently [6% (8/139), *P* < 0.01], though the proportion perceiving themselves as likely to develop tuberculosis in the future was more similar [21% (23/112), *P* = 0.13]. Higher perceived likelihoods of having tuberculosis currently or of developing tuberculosis in the future (among community-enrolled PWTS) and greater inclination toward treatment (among all PWTS) were significantly associated with being started on treatment by six months (*P* = 0.02, 0.02, and 0.03, respectively).

**Table 2:** Baseline survey responses of people with trace sputum stratified by enrollment setting, treatment status by six months, and sex

	Enrollment setting		Treatment status		Sex	
	Community *n* = 129	Clinic *n* = 200	Not treated by 6 months *n* = 178	Treated by 6 months *n* = 151	Male *n* = 193	Female *n* = 136
Reported moderate to high anxiety about possibility of having or developing tuberculosis*, n* [% of column (95%* CI*)]	14 [11% (6–18%)]	52 [26% (20–33%)]	37 [21%, (15–28%)]	29 [19% (13–27%)]	32 [17% (12–23%)]	34 [25% (18–33%)]
Reported inclination towards starting treatment had they not been in a research study,* n* [% of column (95% *CI*)]	67 [52% (43–61%)]	107 [54%, (46–61%)]	84 [47%,( 40–55%)]	90 [60% (51–67%)]	114 [59% (52–66%)]	60/136 [44% (36–53%)]
Perceived themselves to probably or certainly/almost certainly have tuberculosis now, *n* [% of column (95% *CI*)]*	28 [22%, (15–30%)]	*Not asked*	11/77 [14% (8–25%)]	17/52 [33%, (21–47%)]	20/79 [25% (16–37%)]	8/50 [16% (8–30%)]
Perceived themselves as probable or certain/almost certain to develop tuberculosis at some point in the future, *n* [% of column (95% *CI*)]*	30 [23% (16–32%)]	*Not asked*	11/55 [20% (11–33%)]	19/43 [44% (29–60%)]	23/67 [34% (23–47%)]	7/31 [23% (10–42%)]

*CI* = confidence interval

^*^Not asked of participants enrolled from clinic settings

**Fig. 2:**
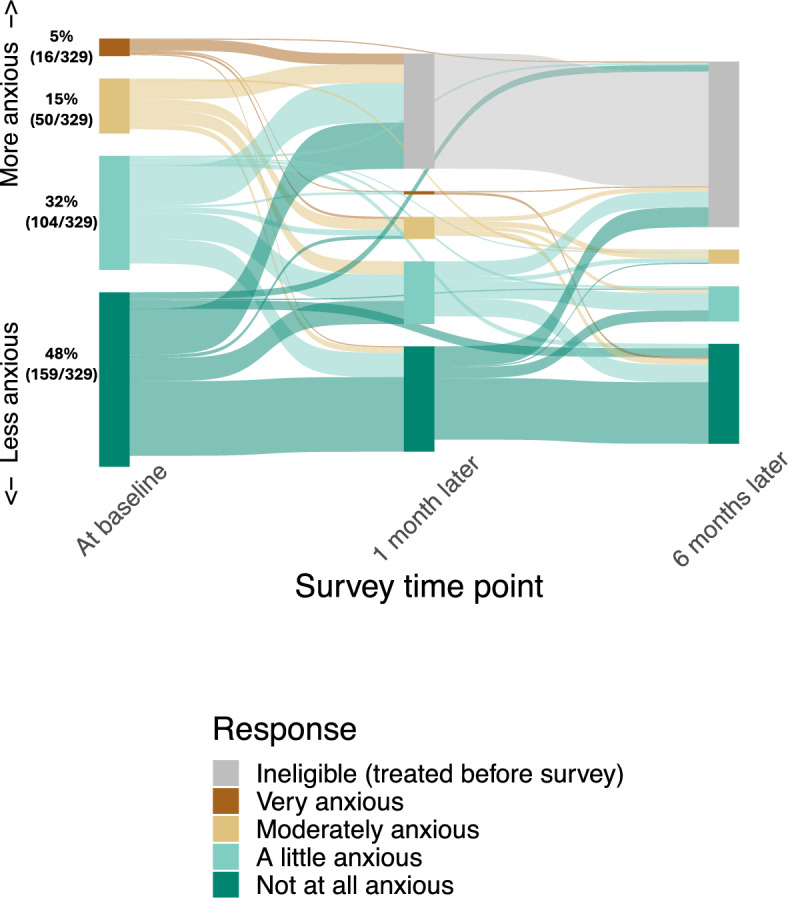
Reported anxiety by participants with a trace Xpert Ultra result, immediately after receiving the result and at one month and six months of follow up. Participants who remained untreated for tuberculosis were asked about the level of anxiety they experienced related to their trace result. Loss to follow up is represented by portions of each vertical bar that do not continue to the right as colored ribbons

One month after enrollment, 32% (105/329) PWTS had started tuberculosis treatment, including 63% (10/16) of those who were very anxious at baseline (Fig. 2). Of those 10, only 3 had microbiological confirmation of disease (compared to 61% of those started on treatment overall). Most who remained off treatment reported either an unchanged (59%, 103/176) or a decreased (24%, 42/176) level of anxiety at one-month follow-up, while 18% (31/176) reported an increase. By six months, two-thirds of participants who remained off treatment reported no anxiety (67%, 91/136), no participants reported feeling very anxious, and only 15 (13%) of the 118 participants who completed both follow up surveys reported a higher level of anxiety at six months than at one month (Fig. 2).

**Fig. 3:**
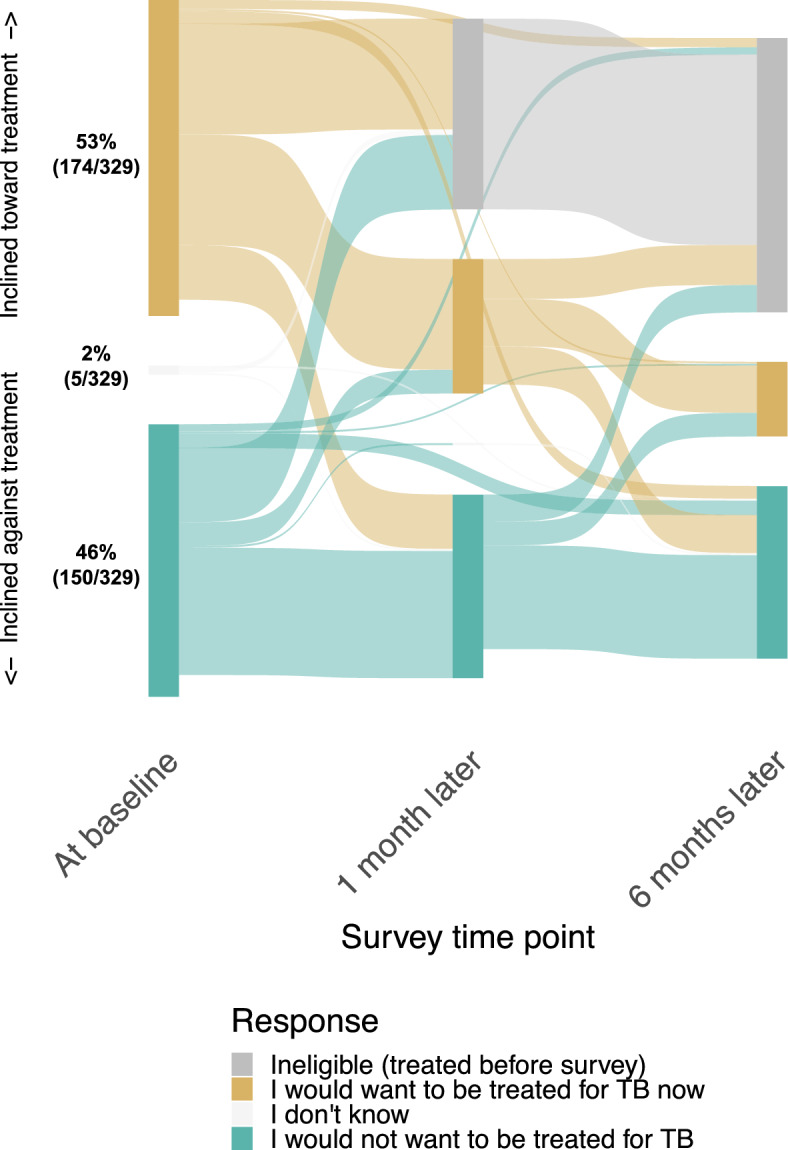
Inclination toward or against tuberculosis (TB) treatment among participants with a trace Xpert Ultra result, immediately after receiving the result and at one month and six months of follow up. Participants who remained untreated for tuberculosis were asked whether they would favor receiving or not receiving treatment, had they not been part of a research study. Loss to follow up is represented by portions of each vertical bar that do not continue to the right as colored ribbons

When asked whether they would be inclined to pursue tuberculosis treatment if they were not being offered monitoring and repeat testing as part of the study, a slight majority of PWTS expressed an inclination toward treatment at baseline (53%, 174/329). Most of those who remained untreated continued to report the same treatment inclination at one month (74%, 131/176) and six months (68%, 93/136) as at baseline, but participants whose opinion changed from baseline to six months were more likely to change toward not wanting tuberculosis treatment (81%, 35/43) than toward favoring tuberculosis treatment (19%, 8/43; *P* < 0.01) (Fig. 3**)**. By six months, fewer favored treatment (30%, 41/136) than at baseline (*P* < 0.01).

### Test sensitivity versus specificity vignette responses

When presented with choices between a more sensitive test and a more specific test, 59% of all participants (503/847) preferred the more sensitive option in the first scenario where this test’s positive predictive value (PPV) was 50% (Table 3). The inclination toward a more sensitive test was stronger among participants recruited from the community setting (70%) than from health facilities (51%; *P* < 0.01). Responses did not vary significantly by initial Xpert Ultra result. In the second scenario in which the “too strong” test had a lower (17%) PPV, the proportion preferring this more sensitive option decreased to 46%.

**Table 3:** Participants’ preferences regarding tuberculosis test sensitivity versus specificity, by enrollment setting and initial Xpert Ultra result

		Scenario 1: More sensitive test (100% sensitivity; 50% PPV) versus more specific test (50% sensitivity; 100% PPV)				Scenario 2^1^**:** More sensitive test (100% sensitivity; 17% PPV) versus more specific test (50% sensitivity; 100% PPV)^2^			
	*n*	Prefer more sensitive test	Prefer more specific test	Prefer no test	*P*-value^3^	Prefer more sensitive test	Prefer more specific test	Prefer no test	*P-*value^3^
All participants	847	503 (59%)	325 (38%)	19 (2%)		383 (46%)	443 (53%)	21 (3%)	
Enrollment Setting					< 0.01				< 0.01
Community	376	263 (70%)	105 (28%)	8 (2%)		202 (54%)	165 (44%)	9 (2%)	
Health facility	471	240 (51%)	220 (47%)	11 (2%)		181 (40%)	278 (61%)	12 (3%)	
Initial Xpert Ultra result					0.34				0.23
Trace	329	202 (61%)	119 (36%)	8 (2%)		146 (45%)	174 (54%)	9 (3%)	
Positive	239	149 (62%)	86 (36%)	4 (2%)		122 (52%)	113 (48%)	4 (2%)	
Negative	279	152 (54%)	120 (43%)	7 (3%)		115 (41%)	156 (56%)	8 (3%)	

*PPV* positive predictive value

^1^Those who preferred the more sensitive test or no test in Scenario 1 were assumed to make the same choice in Scenario 2

^2^In Scenario 2, the more sensitive test is described in the vignette as misdiagnosing 10 people with tuberculosis for every 2 people correctly diagnosed with tuberculosis (see Appendix A)

^3^Pearson’s Chi-squared test comparing enrollment settings or study arms

### Ratings of diagnostic testing

All diagnostic tests used to further evaluate PWTS were rated favorably by participants who had completed them, with nearly all participants reporting that they considered the tests valuable and did not mind completing them (Supplemental Fig. S1).

## Discussion

When managing trace sputum results with an approach of treatment deferral, further diagnostic evaluation, and close monitoring in the context of a research study, we found that most PWTS (78%) did not think they were likely to have tuberculosis or develop it in the future (77%) and 80% reported no or little associated anxiety at the time of enrollment. Over the following six months, 47% initiated treatment, but those who remained off treatment reported further reductions in anxiety and became less likely to favor initiating tuberculosis treatment. Participants also rated their experiences of completing multiple tuberculosis diagnostic procedures quite favorably. Altogether, our findings suggest that further diagnostic evaluation, with close monitoring off treatment for those who do not have other evidence of tuberculosis at baseline, is acceptable to the majority of PWTS.

For those PWTS least likely to have active tuberculosis, a monitoring approach may also minimize harms from overtreatment. PWTS identified through screening, for example, may be less likely to have bacteriologically confirmed tuberculosis; the few studies that have used Xpert Ultra as an initial screening test found positive sputum cultures in only 10–14% of people with trace results [14, 15]. In surveys that screened with symptoms and/or CXR prior to Xpert Ultra testing, the prevalence of culture positivity among those with a trace result was higher though still under 55% [5, 16, 17]. Patients with a history of prior tuberculosis are another population in whom trace results are relatively likely to be false-positive [3, 4, 18]. An early report of longitudinal follow-up of PWTS not treated for tuberculosis suggests that some with negative baseline sputum cultures may still be at elevated risk to develop tuberculosis [19]; however, if the lowest risk PWTS could be reliably identified, they may benefit from treatment deferral.

Ideally, decisions to treat a patient with a trace result would be based on an individual risk assessment followed by shared decision-making that takes both likely outcomes and patient preference into consideration. However, not only is the risk of tuberculosis in PWTS poorly understood, the ideal balance between potential harms of over- and undertreatment of possible tuberculosis is not well established. Surveys of clinicians suggest they may overweight harms of unnecessary tuberculosis treatment, requiring a higher probability threshold (between 20 and 60%) when making treatment decisions intuitively than when guided in systematic estimation of utilities (often leading to treating patients with < 10% probability of tuberculosis) [20]. From the patient perspective, responses to our survey about tradeoffs between test sensitivity versus specificity suggest participants’ intuitive tolerance for overdiagnosis and overtreatment is at least as high as clinicians’, with a majority favoring a tuberculosis test with a PPV of 50% over one that would miss cases. Thus, deferring treatment for PWTS may only be appropriate when the probability of tuberculosis is particularly low, or when close follow-up can ensure that patients who have tuberculosis are highly likely to be identified and treated before they experience harm.

Even when further diagnostic testing and monitoring after a trace result would be acceptable to most patients and clinicians, the resources required may be unavailable. Diagnostic evaluations performed in this study, such as CT scans and repeated cultures, are often performed to assess uncertain tuberculosis status in high-resource settings but may be unavailable or prohibitively expensive in many places with a high burden of tuberculosis. Additionally, without dedicated personnel to follow up with participants, longitudinal monitoring may not be feasible. Importantly, we found that half of the PWTS who consented to the study would have preferred initiating tuberculosis treatment at baseline if they had not been enrolled in the study that provided close monitoring and detailed reassessment, which may not be always available in some routine care settings.

Our study has some limitations. Anxiety or desire to start tuberculosis treatment may have influenced patients’ care-seeking or symptom reporting, biasing which study participants remained untreated and eligible for follow-up surveys. The language used in our questionnaire could also have biased the responses elicited from participants, depending on the translation. Social desirability may have led patients to minimize any negative feelings, especially as the survey was administered by study staff. Participating in research that provided resources for further testing and follow-up may have alleviated anxiety about treatment deferral among PWTS. Finally, while our structured questionnaire facilitated summarization and comparison of responses across a large sample, more in-depth qualitative data would be needed to understand the range of patient experiences.

## Conclusions

In summary, for patients with a trace Xpert Ultra result of uncertain clinical significance, deferring treatment decisions may be acceptable from the patient perspective when further diagnostic evaluation and monitoring can be offered. Tuberculosis treatment decision-making for PWTS should consider individual tuberculosis risk, patient preferences, and available resources to support appropriate additional testing and monitoring. Further studies will be needed to optimize management of PWTS and assess the clinical impact of treatment decision deferral.

## Supplementary Information

Additional file 1. Fig. S1. Ratings of the perceived valuableness and unpleasantness of diagnostic tests completed at baseline by participants with trace Xpert Ultra results. Participants with trace Xpert Ultra results were asked on a survey administered at the one month follow up visit to rate the valuableness and unpleasantness of each diagnostic test completed during the baseline evaluation.

Additional file 2. Appendix A.

## Data Availability

The dataset used during the current study are available on reasonable request. Such requests should be directed to Emily Kendall, MD, PhD at ekendall@jhmi.edu.

## Abbreviations

*CI*: Confidence interval
CRP: C-reactive protein
CT: Computed tomography
CXR: Chest x-ray
IQR: Interquartile range
HIV: Human immunodeficiency virus
LAM: Lipoarabinomannan
MTB: *Mycobacterium tuberculosis*
PPV: Positive predictive value
PWTS: People with trace sputum
Q1: 25th percentile
Q3: 75th percentile
Xpert Ultra: Xpert MTB/RIF Ultra (Cepheid)

## References

[1] Chakravorty S, Simmons A, Rowneki M, Parmar H, Cao Y, Ryan J, et al. The new Xpert MTB/RIF Ultra: improving detection of *Mycobacterium**tuberculosis* and resistance to rifampin in an assay suitable for point-of-care testing. MBio. 2017;8(4):e00812-e817. 28851844 10.1128/mBio.00812-17 PMC5574709

[2] Berhanu RH, David A, da Silva P, Shearer K, Sanne I, Stevens W, et al. Performance of Xpert MTB/RIF, Xpert Ultra, and Abbott RealTime MTB for diagnosis of pulmonary tuberculosis in a high-HIV-burden setting. J Clin Microbiol. 2018;56(12):10. 10.1128/JCM.00560-18 PMC6258835 30305387

[3] Dorman SE, Schumacher SG, Alland D, Nabeta P, Armstrong DT, King B, et al. Xpert MTB/RIF Ultra for detection of *Mycobacterium**tuberculosis* and rifampicin resistance: a prospective multicentre diagnostic accuracy study. Lancet Infect Dis. 2018;18(1):76–84. 29198911 10.1016/S1473-3099(17)30691-6 PMC6168783

[4] Esmail A, Tomasicchio M, Meldau R, Makambwa E, Dheda K. Comparison of Xpert MTB/RIF (G4) and Xpert Ultra, including trace readouts, for the diagnosis of pulmonary tuberculosis in a TB and HIV endemic setting. Int J Infect Dis. 2020;95:246–52. 32247825 10.1016/j.ijid.2020.03.025

[5] Floyd S, Klinkenberg E, de Haas P, Kosloff B, Gachie T, Dodd PJ, et al. Optimising Xpert-Ultra and culture testing to reliably measure tuberculosis prevalence in the community: findings from surveys in Zambia and South Africa. BMJ Open. 2022;12(6): e058195. 35710250 10.1136/bmjopen-2021-058195 PMC9207894

[6] World Health Organization. WHO operational handbook on tuberculosis: module 3: diagnosis—rapid diagnostics for tuberculosis detection. Geneva: World Health Organization; 2021.

[7] Choi H, Park HA, Hyun IG, Kim JH, Hwang YI, Jang SH, et al. Incidence and outcomes of adverse drug reactions to first-line anti-tuberculosis drugs and their effects on the quality of life: a multicenter prospective cohort study. Pharmacoepidemiol Drug Saf. 2022;31(11):1153–63. 35909258 10.1002/pds.5513

[8] Sant Anna FM, Araujo-Pereira M, Schmaltz CAS, Arriaga MB, Andrade BB, Rolla VC. Impact of adverse drug reactions on the outcomes of tuberculosis treatment. PLoS ONE. 2023;18(2): e0269765. 36749743 10.1371/journal.pone.0269765 PMC9904486

[9] Grede N, Claros JM, de Pee S, Bloem M. Is there a need to mitigate the social and financial consequences of tuberculosis at the individual and household level? AIDS Behav. 2014;18(Suppl 5):S542–53. 24710958 10.1007/s10461-014-0732-0

[10] Dodor E. The feelings and experiences of patients with tuberculosis in the Sekondi-Takoradi Metropolitan district: implications for TB control efforts. Ghana Med J. 2012;46:211–8. 23661839 PMC3645176

[11] Patel N, Patel H, Varu J, Gandhi R, Murugan Y. The invisible toll: unveiling the prevalence and predictors of depression and anxiety among pulmonary tuberculosis (TB) patients and their households in Gujarat, India. Cureus. 2024;16(7): e65015. 39165433 10.7759/cureus.65015 PMC11333847

[12] Sutar R, Majumdar A, Yadav V, Basera DS, Gupta H. Anxiety, stress, and quality of life in patients with tuberculosis: A systematic review and meta-analysis. Ind Psychiatry J. 2024;33(1):13–29. 38853803 10.4103/ipj.ipj_58_23 PMC11155636

[13] Steadman A, Andama A, Ball A, Mukwatamundu J, Khimani K, Mochizuki T, et al. New manual quantitative polymerase chain reaction assay validated on tongue swabs collected and processed in Uganda shows sensitivity that rivals sputum-based molecular tuberculosis diagnostics. Clin Infect Dis. 2024;78(5):1313–20. 38306491 10.1093/cid/ciae041 PMC11093664

[14] Sung J, Nantale M, Nalutaaya A, Biche P, Mukiibi J, Kamoga CE, et al. Evidence for tuberculosis in individuals with Xpert Ultra “trace" sputum during screening of high-burden communities. Clin Infect Dis. 2024;78(3):723–9. 37787077 10.1093/cid/ciad595 PMC10954329

[15] Berhanu RH, Lebina L, Nonyane BAS, Milovanovic M, Kinghorn A, Connell L, et al. Yield of facility-based targeted universal testing for tuberculosis with Xpert and mycobacterial culture in high-risk groups attending primary care facilities in South Africa. Clin Infect Dis. 2023;76(9):1594–603. 36610730 10.1093/cid/ciac965 PMC10156124

[16] Moyo S, Ismail F, Van der Walt M, Ismail N, Mkhondo N, Dlamini S, et al. Prevalence of bacteriologically confirmed pulmonary tuberculosis in South Africa, 2017–19: a multistage, cluster-based, cross-sectional survey. Lancet Infect Dis. 2022;22(8):1172–80. 35594897 10.1016/S1473-3099(22)00149-9 PMC9300471

[17] Matji R, Maama L, Roscigno G, Lerotholi M, Agonafir M, Sekibira R, et al. Policy and programmatic directions for the Lesotho tuberculosis programme: findings of the national tuberculosis prevalence survey, 2019. PLoS ONE. 2023;18(3): e0273245. 36893175 10.1371/journal.pone.0273245 PMC9997977

[18] Mishra H, Reeve BWP, Palmer Z, Caldwell J, Dolby T, Naidoo CC, et al. Xpert MTB/RIF Ultra and Xpert MTB/RIF for diagnosis of tuberculosis in an HIV-endemic setting with a high burden of previous tuberculosis: a two-cohort diagnostic accuracy study. Lancet Respir Med. 2020;8(4):368–82. 32066534 10.1016/S2213-2600(19)30370-4

[19] Sung J, Nantale M, Nalutaaya A, Biché P, Mukiibi J, Akampurira J, et al. The long-term risk of tuberculosis among individuals with Xpert Ultra “trace” screening results: a longitudinal follow-up study. medRxiv. 2025. 10.1101/2025.03.20.25324205.40492100

[20] de Rooij ML, Lynen L, Decroo T, Henriquez-Trujillo AR, Boyles T, Jacobs BKM. The therapeutic threshold in clinical decision-making for TB. Int Health. 2023;15(6):615–22. 36744621 10.1093/inthealth/ihad002 PMC10629962

