# Supplementary material for: Patient experiences of tuberculosis treatment deferral after a trace Xpert Ultra result: a prospective cohort study
Source: Infect Dis Poverty. 2025 Jul 15;14:68. doi: 10.1186/s40249-025-01338-0 (PMC12261543; doi:10.1186/s40249-025-01338-0)
Supplement: Supplementary file 1 — Additional file 1. Fig. S1. Ratings of the perceived valuableness and unpleasantness of diagnostic tests completed at baseline by participants with trace Xpert Ultra results. Participants with trace Xpert Ultra results were asked on a survey administered at the one month follow up visit to rate the valuableness and unpleasantness of each diagnostic test completed during the baseline evaluation. [file 40249_2025_1338_MOESM1_ESM.pdf]

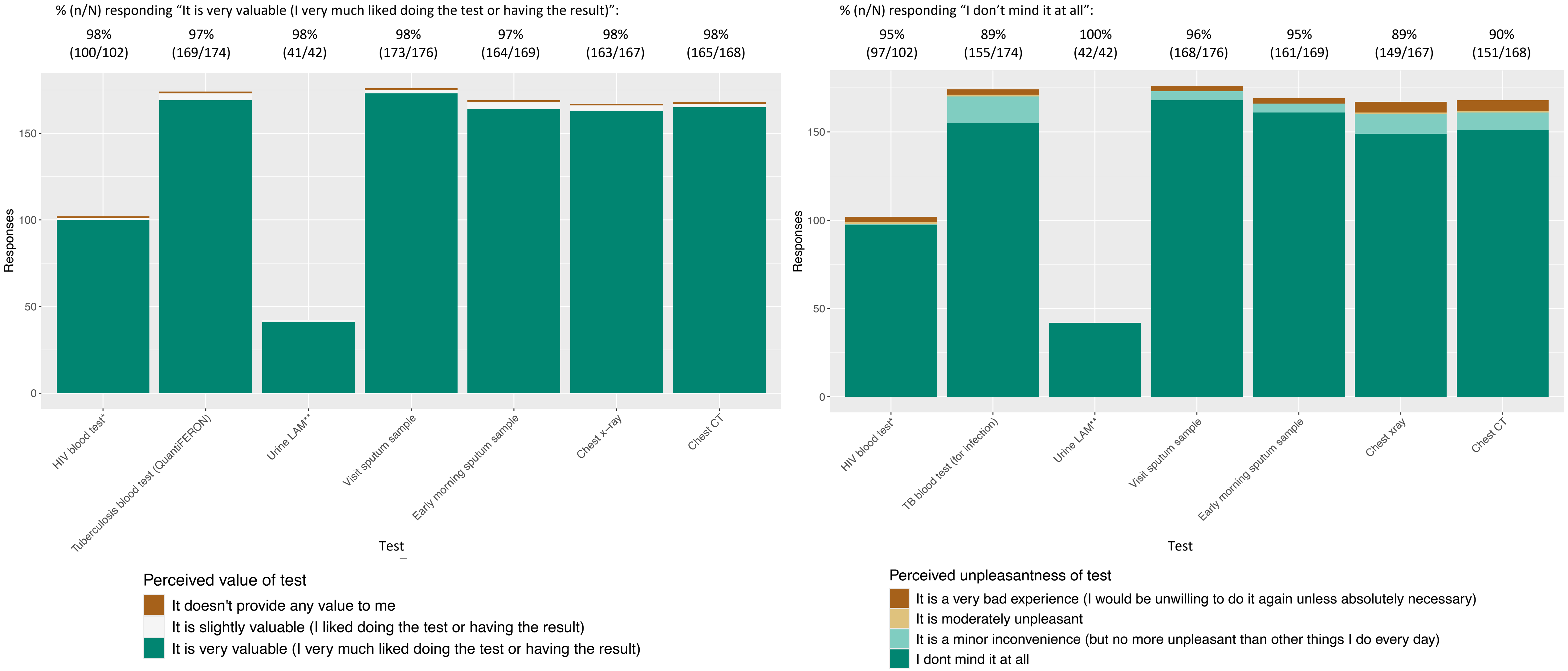

LAM: Lipoarabinomannan; CT: computed tomography
*For patients without known HIV only
**For patients with HIV only
Number of responses differ by test as ratings only included for participants who had completed that test during the baseline evaluation.
