# Supplementary material for: Patient experiences of tuberculosis treatment deferral after a trace Xpert Ultra result: a prospective cohort study
Source: Infect Dis Poverty. 2025 Jul 15;14:68. doi: 10.1186/s40249-025-01338-0 (PMC12261543; doi:10.1186/s40249-025-01338-0)
Supplement: Supplementary file 2 — Additional file 2. Appendix A. [file 40249_2025_1338_MOESM2_ESM.docx]

Appendix A. Preference Interview Instrument

**Part 1. Subjective questions about TB screening participation and results *[community screening participants only]***

1. You were invited to enroll in this study because of a TB test that you recently did when we were testing people in your community. In your own words, how would you explain the result of your test to a friend or family member?

2. Why did you choose to participate in TB screening? Choose the reason that best explains your participation.

1. I wanted to make sure I was healthy
2. I was worried I might have TB
3. My family, friends, or neighbors were participating
4. I heard about the testing from someone and wanted to check it out
5. The study staff asked me to test and I didn't feel I could say no
6. I was passing by and wanted to see what was going on
7. Other (specify in comment)

3. Before you were tested in your community, what did you think about whether you had TB?

1. I was certain I did not have TB
2. I thought I probably did not have TB
3. I was worried I might have TB
4. I thought I probably did have TB
5. I was certain or almost certain I had TB
6. I hadn't thought about it or didn't have an opinion

4. Before you were tested in your community, what did you think about whether you had TB?

1. I certainly don't have TB
2. I probably don't have TB
3. I'm worried I might have TB
4. I think I probably do have TB
5. I am certain or almost certain I have TB

4.1 Before you were tested in your community, what did you think about whether you had TB?

1. I certainly won't develop TB
2. I probably won't develop TB
3. I'm worried I might develop TB
4. I think I will probably develop TB
5. I am certain or almost certain I will develop TB

**Part 2. Ratings of diagnostic tests**

***[trace participants and negative controls]***

We have asked you to complete several types of tests to determine whether you have TB. We will ask you about your experience of these tests: first about the negative part of the experience (difficulty, discomfort, of inconvenience), and then about any positive part of the experience (any value of enjoyment). Please answer based on your experience -- or any tests you didn't do, answer based on what you think the experience would have been if you had done the test).

First, please rate how unpleasant (how difficult, uncomfortable, or inconvenient) each test is.

1) Blood tests for TB

1. I don't mind it at all
2. It is a minor inconvenience (but no more unpleasant than other things I do every day)
3. It is moderately unpleasant
4. It is a very bad experience (I would be unwilling to do it again unless absolutely necessary)
5. I don't know/can't answer

2) Blood tests for HIV

1. I don't mind it at all
2. It is a minor inconvenience (but no more unpleasant than other things I do every day)
3. It is moderately unpleasant
4. It is a very bad experience (I would be unwilling to do it again unless absolutely necessary)
5. I don't know/can't answer

3) Sputum test collected during a study visit ("spot" sputum)

1. I don't mind it at all
2. It is a minor inconvenience (but no more unpleasant than other things I do every day)
3. It is moderately unpleasant
4. It is a very bad experience (I would be unwilling to do it again unless absolutely necessary)
5. I don't know/can't answer

4) Early morning sputum test

1. I don't mind it at all
2. It is a minor inconvenience (but no more unpleasant than other things I do every day)
3. It is moderately unpleasant
4. It is a very bad experience (I would be unwilling to do it again unless absolutely necessary)
5. I don't know/can't answer

5) Smartphone cough monitor for 2 days and nights

1. I don't mind it at all
2. It is a minor inconvenience (but no more unpleasant than other things I do every day)
3. It is moderately unpleasant
4. It is a very bad experience (I would be unwilling to do it again unless absolutely necessary)
5. I don't know/can't answer

6) Chest X-ray

1. I don't mind it at all
2. It is a minor inconvenience (but no more unpleasant than other things I do every day)
3. It is moderately unpleasant
4. It is a very bad experience (I would be unwilling to do it again unless absolutely necessary)
5. I don't know/can't answer

7) Chest CT

1. I don't mind it at all
2. It is a minor inconvenience (but no more unpleasant than other things I do every day)
3. It is moderately unpleasant
4. It is a very bad experience (I would be unwilling to do it again unless absolutely necessary)
5. I don't know/can't answer

8) Urine test for TB

1. I don't mind it at all
2. It is a minor inconvenience (but no more unpleasant than other things I do every day)
3. It is moderately unpleasant
4. It is a very bad experience (I would be unwilling to do it again unless absolutely necessary)
5. I don't know/can't answer

Now please rate any positive aspects of the experience. How valuable was each of these tests for you (or how valuable to you think it would be if you had done it)?

9) Blood tests for TB

1. It doesn't provide any value to me
2. It is slightly valuable (I liked doing the test or having the result)
3. It is very valuable (I very much liked doing the test or having the result)
4. I don't know/can't answer

10) Blood tests for HIV

1. It doesn't provide any value to me
2. It is slightly valuable (I liked doing the test or having the result)
3. It is very valuable (I very much liked doing the test or having the result)
4. I don't know/can't answer

11) Sputum test collected during a study visit ("spot" sputum)

1. It doesn't provide any value to me
2. It is slightly valuable (I liked doing the test or having the result)
3. It is very valuable (I very much liked doing the test or having the result)
4. I don't know/can't answer

12) Early morning sputum test

1. It doesn't provide any value to me
2. It is slightly valuable (I liked doing the test or having the result)
3. It is very valuable (I very much liked doing the test or having the result)
4. I don't know/can't answer

13) Smartphone cough monitor for 2 days and nights

1. It doesn't provide any value to me
2. It is slightly valuable (I liked doing the test or having the result)
3. It is very valuable (I very much liked doing the test or having the result)
4. I don't know/can't answer

14) Chest X-ray

1. It doesn't provide any value to me
2. It is slightly valuable (I liked doing the test or having the result)
3. It is very valuable (I very much liked doing the test or having the result)
4. I don't know/can't answer

15) Chest CT

1. It doesn't provide any value to me
2. It is slightly valuable (I liked doing the test or having the result)
3. It is very valuable (I very much liked doing the test or having the result)
4. I don't know/can't answer

16) Urine test for TB

1. It doesn't provide any value to me
2. It is slightly valuable (I liked doing the test or having the result)
3. It is very valuable (I very much liked doing the test or having the result)
4. I don't know/can't answer

17) Comments/notes on participants' perception of tests' burden or value

**Part 3. Vignette for developing future discrete choice experiment**

***[all participants]***

I want you to consider an imaginary situation. Imagine that we are planning to test your friends and family for tuberculosis.

The goal of testing is to find people who have TB, so that they can be treated. If people who have TB don't get treated, they can become very sick, sometimes die, and spread TB to others.

But we want to avoid treating people who don't have TB, because treatment requires 6 months of taking several medicines every day, and people often find this unpleasant or disruptive.

Imagine that there are two tests that could be used to test your family and friends for tuberculosis.

One test is "too strong". It will be positive for everyone who has TB, so that they can get treated and be cured. But it will also be positive for some people who don't have TB. Every time the strong test correctly finds someone who really has TB, it will also tell one person without TB that they have TB and need treatment.

The other test is "too weak". If it says that someone has TB, it will be correct. But it will miss one out of every two people who have TB, so they won't be able to get treated before they get sick or spread infection to others.

1) If you could only recommend that your family and friends get tested with one of these two tests, which test would you choose?

- 1. The test that is too strong
  2. The test that is too weak
  3. No test

2) You chose the test that is too strong. What is the most important reason why you chose the too-strong test?

1. I wouldn't want my friends and family to get sick with TB.
2. I wouldn't want people with TB to spread it to others.
3. I wouldn't want people who really have TB to be told that they don't have it.
4. Getting treatment for TB is not a big deal.
5. Other

3) Specify other reason for choosing too-strong test.

4) Now imagine that the too-strong test is a little different. In order to find the two (2) people who have TB, ten (10) other people who don't have TB will test positive and get treatment that they don't need.

Would you still choose the too-strong test?

- 1. Yes, I would still choose the too-strong test
  2. No, I would choose the test that is too weak
  3. No, I would choose no test

5) You chose the test that is too weak. What is the most important reason why you chose the too-weak test?

1. I don't think a person who doesn't have TB should be told that they have TB.
2. The other person who has TB can wait to get treated once he/she becomes sick.
3. TB treatment is difficult or unpleasant.
4. TB treatment might cause harm to the person who doesn't need it.
5. Other

6) Specify other reason for choosing too-weak test

7) Why would you not want your family and friends to take either test?

1. It's better not to get tested at all than to risk getting an incorrect result.
2. I don't think any of them could have TB.
3. Neither test seems good enough, so they should wait for a better option or wait to see if they get sick.
4. Other

8) Specify other reason for choosing no test.

**Part 4. More questions about trace positive experience *[participants with trace positive results only]***

We have not recommended that you start treatment right now, because based on the information that we have so far, it is more likely than not that you do not have TB. But we are asking you to do additional tests (sputum tests, blood tests, x-rays) and follow-up visits to determine whether you have TB and should be treated.

1) How do you feel about the plan to do more tests and monitoring to determine whether you need treatment?

1. I am very dissatisfied
2. I am somewhat dissatisfied
3. I am neither satisfied nor dissatisfied
4. I am somewhat satisfied
5. I am very satisfied
6. Unable/unwilling to answer

2) [If somewhat or very dissatisfied] Why are you dissatisfied (check all that apply)

1. I don't like the uncertainty of not knowing whether I have TB
2. I don't like doing so much testing
3. I don't think I could have TB.
4. Other
5. Don't know / don't want to answer

3) Specify other reason for dissatisfaction.

4) How anxious do you feel about the possibility that you may have TB or may develop TB?

1. Not at all anxious
2. A little anxious (I don't like the uncertainty, but it won't affect my state of mind)
3. Moderately anxious (I will probably worry about this from time to time)
4. Very anxious (This will cause me severe or frequent stress)
5. Unable/unwilling to answer

5) Based on what you have learned about your TB test result, if we weren't offering you additional testing in this research study, would you seek out TB treatment at this time?

1. I would want to be treated for TB now
2. I would not want to be treated for TB
3. I don't know

6) In a few words, please explain why you would or would not want to be treated.
